# Direct quantification of changes in pH within single levitated microdroplets and the kinetics of nitrate and chloride depletion†

**DOI:** 10.1039/d2sc06994f

**Published:** 2023-04-11

**Authors:** Kyle J. Angle, Vicki H. Grassian

**Affiliations:** a Department of Chemistry and Biochemistry, University of California San Diego La Jolla CA 92093 USA vhgrassian@ucsd.edu

## Abstract

The hygroscopicity and pH of aqueous microdroplets and smaller aerosols control their impacts on human health and the climate. Nitrate depletion and chloride depletion through the partitioning of HNO_3_ and HCl into the gas phase are processes that are enhanced in micron-sized and smaller aqueous droplets and this depletion influences both hygroscopicity and pH. Despite a number of studies, uncertainties remain about these processes. While acid evaporation and the loss of HCl or HNO_3_ have been observed during dehydration, there is a question as to the rate of acid evaporation and whether this can occur in fully hydrated droplets at higher relative humidity (RH). To directly elucidate the kinetics of nitrate and chloride depletion through evaporation of HNO_3_ and HCl, respectively at high RH, single levitated microdroplets are probed with cavity-enhanced Raman spectroscopy. Using glycine as a novel *in situ* pH probe, we are able to simultaneously measure changes in microdroplet composition and pH over timescales of hours. We find that the loss of chloride from the microdroplet is faster than that of nitrate, and the calculated rate constants infer that depletion is limited by the formation of HCl or HNO_3_ at the air–water interface and subsequent partitioning into the gas phase.

## Abstract 2

Glycine is used as an *in situ* probe of the kinetics of changes in aqueous microdroplet pH as chloride and nitrate deplete within the microdroplet due to the protonated strong acid forming at the air/water interface and partitioning into the gas phase.

## Graphical abstract

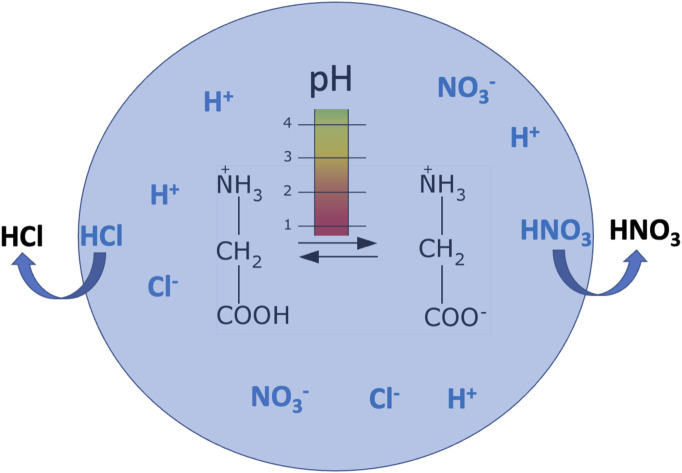

## Introduction

Aqueous microdroplets and smaller aqueous aerosols are ubiquitous in the atmosphere and have profound effects on the global climate and human health. The chemical composition of these aqueous particles often includes an organic fraction as well as salts such as nitrates, chlorides and sulfates. The composition of the particle determines its hygroscopicity, which in turn controls its size at a given relative humidity.^1,2^ Another important aqueous parameter, acidity, can influence cloud condensation nucleus activity, surface chemistry, and interactions with the lungs.^3–5^ Chemical composition and pH are both sensitive to multiphase buffering, as acids and bases can partition out into the surrounding environment.^6^

Two particular types of processes, nitrate depletion and chloride depletion due to the evaporation of the protonated strong acids, HNO_3_ and HCl, respectively, are highly important to the pH of the droplet. These processes are shown in eqn (1) through (4).

1 HA (aq) + NO_3_^−^ (aq) ⇌ HNO_3_ (aq) + A^−^ (aq)

2 HA (aq) + Cl^−^ (aq) ⇌ HCl (aq) + A^−^ (aq)

3 HNO_3_ (aq) ⇌ HNO_3_ (g)

4 HCl (aq) ⇌ HCl (g)

In the first two reactions, (1) and (2), HA is generally used to refer to other acid molecules, often organic acids. These processes taken together are hereafter termed “depletion”. The equilibria shown in eqn (1) and (2) typically lie to the left in bulk solutions since HNO_3_ and HCl are strong acids. However, both of these acids are highly volatile and therefore likely to partition into the surrounding atmosphere once formed at the air/water interface. While the equilibria written in (3) and (4) are controlled by Henry's law, the kinetics of acid evaporation can vary as they depend on several other factors including overall composition.^7^

Depletion of chloride and nitrate in aqueous droplets has been observed in various field and laboratory studies, and occurs to a great enough extent to impact hygroscopicity and source apportionment considerations.^2,8–11^ However, several fundamental aspects of the depletion process remain unclear. Since HNO_3_ or HCl must form at or diffuse to the surface in order to partition into the gas phase, one might assume that depletion would be greatest for smaller aqueous droplets with higher surface area to volume ratios. Indeed, differences in depletion between sub- and super-micron sized atmospheric aerosols have been observed.^12^ One important consideration for the size-dependence of depletion is that depletion can create a negative feedback loop. As depletion occurs, pH is increased and fewer nitrate or chloride ions remain, presumably decreasing the probability of the protonated acids, HNO_3_ or HCl, forming. Therefore, the extent of depletion observed in field studies may vary depending on the initial amount of nitrate or chloride salts contained in the particle, which would vary with size. However, two reports from Laskin and co-workers have shown no statistically significant size-dependence for substrate-deposited aerosols.^13,14^ While most studies have focused on small aerosols, there have also been observations of larger rainwater droplets containing less chloride than the corresponding bulk solutions.^15^ Furthermore, organic coatings could inhibit depletion, preventing complete depletion from occurring even for long atmospheric lifetimes.^11,16^ Detailed studies on the kinetics of depletion could resolve complications relating to size-dependence.

An additional area of ongoing investigation is the cause of depletion. In some studies, depletion is attributed to inorganic acids such as sulfuric acid (*i.e.* chloride or nitrate is replaced by sulfate).^17^ Other reports have indicated that dicarboxylic acids can account for a significant fraction of depletion.^18,19^ Acetic acid, by contrast, has been associated with little depletion.^9^ Another mechanism involves joint loss with ammonia.^20^ A further complication is that chloride depletion can be caused by other chemical reactions including reactions with ozone and the OH radical.^21^ In general, aqueous microdroplets contain numerous components, making identification of the drivers of depletion challenging. For field studies, coalescence and agglutination of droplets add yet another degree of complexity to tracking chloride or nitrate depletion.^8^ For laboratory-generated microdroplets and aerosols that are deposited for days before analysis, the volatilization of different components or the action of dehydration could convolute the analysis of these processes.

Although discussions about the stability of protonated forms of strong acids at the air/water interface have been ongoing for some time, these studies are typically carried out using calculations or bulk water.^22–24^ Our studies discussed here show that when the surface-to-volume ratio is high and there is no bulk reservoir, as found for microdroplets and smaller aerosols, the protonated acid form rapidly partitions from the aqueous phase into the gas phase. In this study, we focus on several unique aspects of the evolution of microdroplet composition and pH under humid conditions. First, we show for the first time how glycine can be used as a probe of pH of a microdroplet and how microdroplet pH evolves over time. Second, these studies provide insights and timescales on nitrate and chloride depletion for optically levitated microdroplets at relatively high relative humidity. Third, we have monitored the changes in size at a given relative humidity as droplet composition and pH change. Overall, these different key aspects are highlighted to provide important findings for aqueous microdroplet composition and pH evolution.

## Experimental methods

All samples were created using milliQ water with resistivity > 18.1 MΩ. Sodium chloride and sodium sulfate (both Certified ACS, Fisher Chemical) were baked at 200 °C in an oven for at least 48 hours to help remove organic impurities. Glycine (>98.5%, Fisher BioReagents) and sodium nitrate (>99.0%, Sigma-Aldrich) were used without additional purification. Adjustments to pH were made with concentrated sulfuric acid (96% in water, Thermo Scientific), and the pH of bulk solutions was measured with a pH meter (OAKTON Instruments). Concentrations are given in terms of molality, *m*, (mol kg^−1^). Bulk-phase refractive index measurements were performed using an ABBE-3L refractometer (Bausch & Lomb) as previously described.^25^ The refractive indices of glycine solutions are shown in ESI Fig. S1.†

**Fig. 1:**
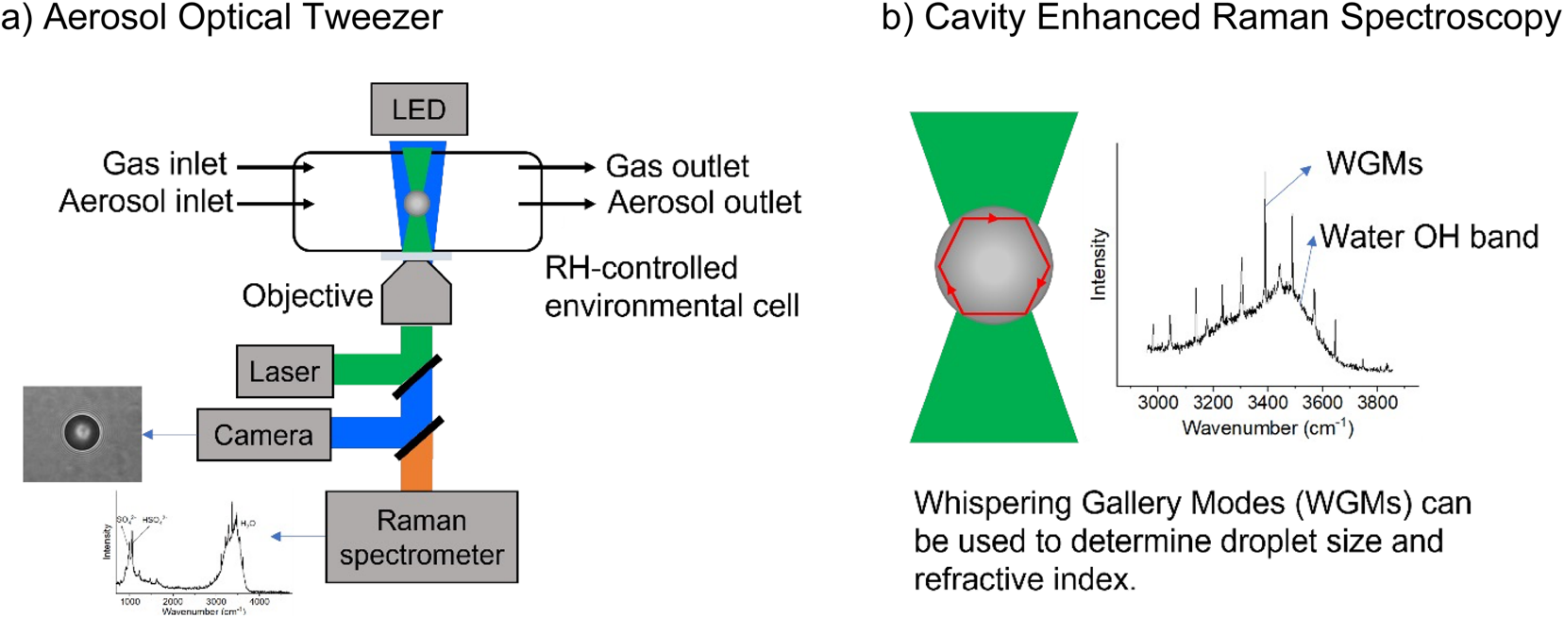
Schematic illustrating (a) the aerosol optical tweezer (AOT) setup and (b) the cavity enhanced Raman spectroscopy.

A commercial Aerosol Optical Tweezer (AOT) 100 from Biral Inc. was used as previously described.^26^ A schematic of the instrument is shown in Fig. 1.^27^ Briefly, microdroplets were formed using an ultrasonic nebulizer (MicroAIR U22, OMRON) resulting in an aqueous droplet *ca.* 3.5 ± 1 microns in radius. Relative humidity (RH) was typically maintained at 80 ± 8% using a controlled flow of wet and dry nitrogen gas (total flow 30 sccm). We note that nebulization forms microdroplets with concentrations that can differ from the bulk solution due to several different factors including water loss between droplet generation and laser trapping. This is discussed more in the ESI.†

The trapping laser had a wavelength of 532 nm and was operated at 50 mW. Raman spectra were acquired every second at 1200 g mm^−1^ setting, with the grating centered either at 570 nm to track the glycine, sulfate, and nitrate peaks, or at 645 nm to track size and refractive index. Each Raman peak location is reported at the same frequency between different experiments; however, Raman maxima for a given experiment shift in the order of ±2 to 4 cm^−1^ due to differences in the chemical matrix and instrument resolution.

While a spherical microdroplet is optically suspended, light of particular frequencies can internally reflect along the particle and constructively interfere with itself, leading to an enhanced Raman signal. These are known as Mie resonances and have also been termed whispering gallery modes (WGMs) due to the similarity of the physics to the phenomenon that causes sound waves to constructively interfere at St. Paul's Cathedral. WGMs are useful as the spacing between them allows calculation of the microdroplet's radius, *r*, and refractive index, *n*_p_, as given in eqn (5),^28^

5 2π*n*_p_*r* = *λm*_w_,

where *λ* is the wavelength of the light circling within the optical cavity and constructively interfering with itself and *m*_w_ is an integer. From multiple WGMs, *n*_p_ and *r* can be simultaneously determined with the precision set using the uncertainty in WGM position.

For these modes to be apparent, the spherical particle must be maintained in the same position in the laser. Small departures in sphericity and equilibrium trapping position, however, can alter the intensity of the WGMs, resulting in WGM occurrences being visually absent from a figure when many spectra are averaged. In some cases, when the traces are all averages of the same number of spectra the relative intensity of WGMs varies from trace to trace.

Additional analyses and calculations required parameters from the Extended Aerosol Inorganics Model (E-AIM).^29,30^ For these calculations, malonic acid was used in place of glycine. This is because these molecules have similar p*K*_a1_ values and calculations with glycine are not feasible. In the E-AIM, organics may be input as acids or bases. An acid is neutral at low pH and an anion at high pH. This does not work for glycine, since it is a cation at low pH, so a neutral E-AIM “glycine” would be improperly charge balanced. On the other hand, a base is neutral at high pH and a cation at low pH. Glycine cannot be input as a base, however, because the first pK_a_ of glycine falls outside the possible range of amine dissociation constants permissible by the E-AIM. Therefore, we used malonic acid instead. Other than this substitution, the E-AIM is run at 80% RH and 298 K.

## Results and analysis

### Glycine as a probe of microdroplet pH

Conjugate acid/base pairs can be used to monitor pH *via* Raman spectral peaks for substrate deposited droplets. Previously, Craig *et al.* employed various conjugate acid/base pairs for pH measurement in substrate-deposited micron-sized droplets.^31^ Among the possibilities given at acidic pH were acetic acid, oxalic acid, bisulfate, and nitric acid itself. In past work, we have seen only weak, diffuse spectral signatures of bisulfate in the AOT spectra.^32^ Oxalic acid generates significantly overlapping Raman peaks and has relatively poor solubility despite its small size.^31,33^ Finally, acetic acid is highly volatile and likely to evaporate from aqueous microdroplets and smaller aqueous aerosols.^34^ To avoid these issues, we used glycine, an amino acid with high solubility and distinct Raman peaks, that would be charged in both its protonated and deprotonated forms so that it would not partition from the microdroplet.

**Fig. 2:**
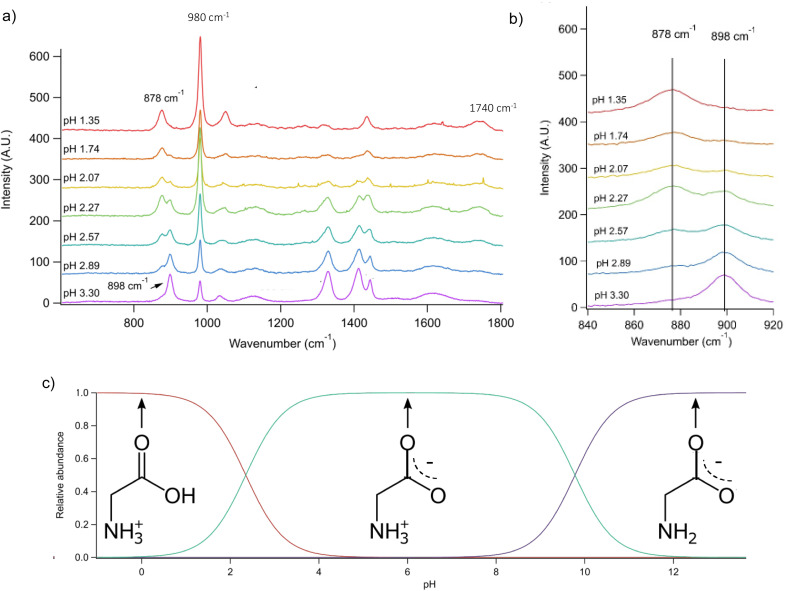
(a) Raman spectra of microdroplets produced from bulk solutions of varying pH. Bulk solutions were acidified by adding an appropriate amount of H_2_SO_4_, so the sulfate peak (980 cm^−1^) is more intense at lower pH. Upon nebulization of a bulk aqueous solution and droplet trapping, minimal acidification is expected as the solutions are already more acidic than CO_2_ and are buffered using 1 m glycine. As pH increases, the intensity of the *ν*(C–COOH) peak at 878 cm^−1^ decreases and the intensity of the *ν*(C–COO^−^), at 898 cm^−1^ increases. The 1740 cm^−1^ peak due to the C=O in the protonated carboxylic acid is also noted and is most prevalent at low pH as expected based on glycine speciation. Traces show the average of 100 spectra. (b) An expanded region of the spectrum showing the changes in peak intensity for the 878 and 898 cm^−1^ that are used for calibration (see the text for further details and Fig. 3). (c) Speciation diagram of glycine showing the relative abundance of the cation (red), the zwitterion (teal), and the anion (violet).

We prepared an AOT calibration curve of glycine microdroplets at various pH levels as shown in Fig. 2. The annotated spectra list pH values corresponding to the bulk solutions from which the glycine-containing droplets were generated. We anticipate that these pH values are close but not identical to the pH of the aqueous solution as we have previously seen acidification upon nebulization for microdroplets by *ca.* 0.1 to 0.3 pH units.^26^ However, since these droplets are already at an acidic pH (eliminating titration by CO_2_) and are airborne for a short enough time, acidification by other trace gases will be minimal, although some decrease in pH may occur due to water evaporation. To help avoid excessive water loss, we maintained RH just above 85%.

Raman spectra of glycine at varying pH are shown in Fig. 2a and an expanded spectral region from 840 to 920 cm^−1^ in Fig. 2b. The spectra show that the ratio of the 878 and 898 cm^−1^ peaks changes as a function of pH and can be used to monitor microdroplet acidity. These two peaks correspond to the C–C stretch in glycine in the cationic (fully protonated) and zwitterionic forms and are labeled as *ν*(C–COOH) and *ν*(C–COO^−^), respectively.^35^ These two forms are shown in the speciation diagram of glycine as a function of pH (Fig. 2c). Additionally, the peak at 1740 cm^−1^ only seen at low pH is due to the C=O stretch of the protonated carboxylic acid group.

The ratio of the intensities of the *ν*(C–COOH) and *ν*(C–COO^−^) peaks within the droplet can be used to calculate acidity and pH as shown in eqn (6):6
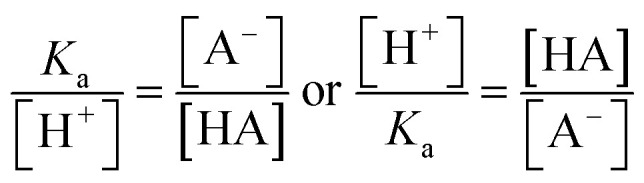


**Fig. 3:**
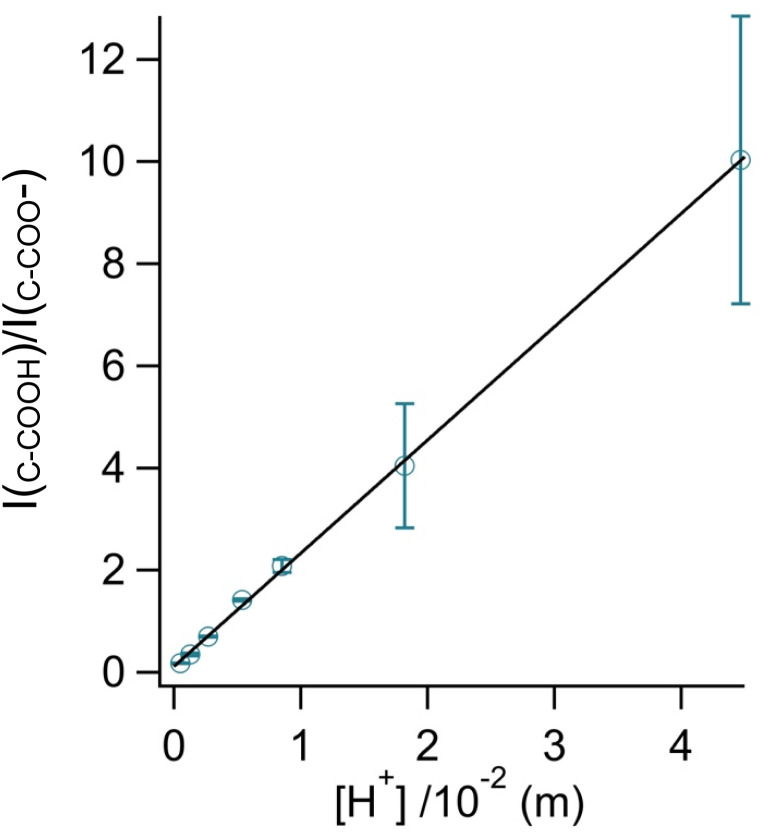
Calibration curve relating the peak intensity ratio of the C–C stretch for the cationic (fully protonated) and zwitterionic forms, 
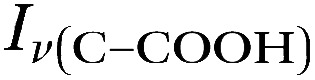
 at 878 cm^−1^ to 
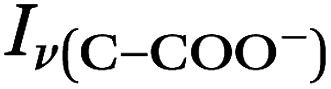
 at 898 cm^−1^, respectively, to determine the H^+^ concentration from the pH values given in Fig. 2.

By rearranging the acid dissociation constant, *K*_a_, the [H^+^] is linearly proportional to the ratio [HA]/[A^−^] which is measured using the relative intensities, *I*_*ν*(C–COOH)_:*I*_*ν*(C–COO^−^)_. This analysis is plotted in Fig. 3. Although individual traces are shown, the data shown in Fig. 3 represent triplicate measurements performed at each pH. Bulk solution phase data also show a linear relationship between the peak ratios and [H^+^] concentration in this pH range from 1.35 to 3.30 confirming the utility of this analysis.

Since we are determining pH from Raman spectral intensities, it is not necessary to select an activity coefficient convention. By assuming that our peak ratios are indicators of the activity of glycine and glycinium, we then know the activity of H^+^, assuming the bulk value of *K*_a1_. Therefore, the definition of pH in this study is governed by the behavior of the ratio of this acid/base pair and may deviate some from pH determined by potentiometric titration, indicator paper, models using Debye–Huckel activity coefficients, or other methods.

### Monitoring of chloride and nitrate depletion within microdroplets and simultaneous changes in pH

With the pH-dependence of the glycine peaks established, experiments to monitor changes in pH and composition through nitrate and chloride depletion through partitioning of HNO_3_ and HCl, respectively, into the gas phase were explored. For these experiments, Raman spectra from single microdroplets containing a mixture of nitrate, glycine, and sulfate (as an internal standard) were collected as a function of time. The nitrate (NO_3_^−^) ion symmetric stretch has a strong Raman signal at 1050 cm^−1^, while sulfate (SO_4_^2−^) has a peak at *ca.* 980 cm^−1^ also assigned to the symmetric stretch. The sulfate peak is not expected to deplete over time, since it is in equilibrium with HSO_4_^−^, which is also a charged species. Sulfuric acid itself is highly acidic, more soluble, and is less volatile than HCl and HNO_3_.^36^ The sulfate peak could increase in intensity, however, if the starting pH was below 3 and pH increases, as bisulfate converts to sulfate. This pH increase could come from nitrate depletion, which would lead to a decrease in the nitrate peak relative to the sulfate peak. We note that the loss of bisulfate in a bulk solution would lead to a decrease in intensity in the 1050 cm^−1^ region; however, in AOT experiments we have observed the bisulfate signal to be weak and diffuse and therefore only a minor contributor to the overall intensity in that spectral region.^32^

**Fig. 4:**
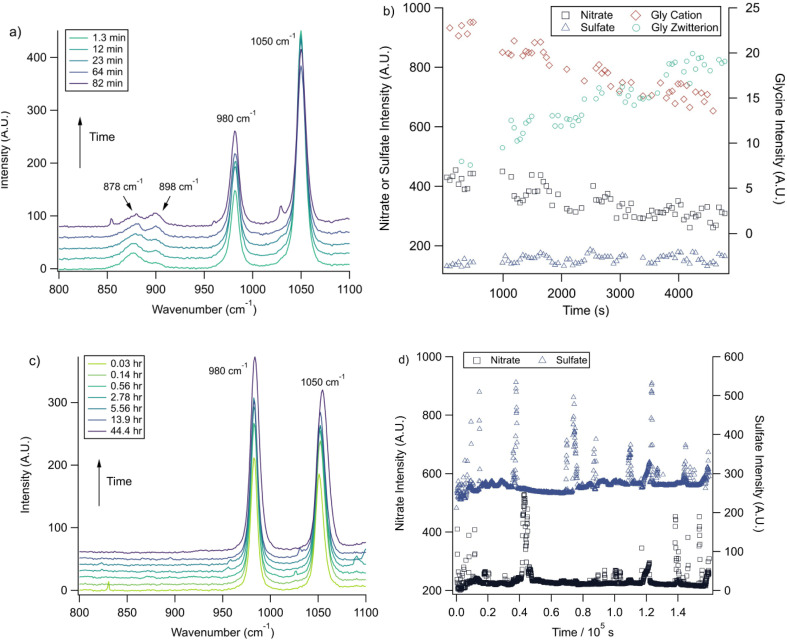
(a) A microdroplet was trapped from a solution of 2 m glycine and 0.1 m NaNO_3_ titrated to pH 1.8 with sulfuric acid. These Raman spectra show, *via* changes in the ratio of the intensities of glycine peaks, 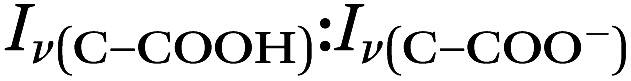, that the pH increases over time while the nitrate peak (1050 cm^−1^) also decreases. (b) Intensity values for these different peaks and the 980 cm^−1^ peak due to sulfate from this microdroplet are plotted as a function of time. Note the separate *y*-axes used to show the changes in the glycine cation and zwitterion peaks compared to the inorganic ion peaks. Error bars are omitted for visual clarity and are typically less than 10% of the data point value. (c) A microdroplet was trapped from a solution of 1 m NaNO_3_ and 1 m Na_2_SO_4_ at higher pH (*ca.* 5.4). The change in the nitrate and sulfate peak intensities over short and long time periods (up to 44 hours) varied very little indicating that no nitrate depletion occurred in this microdroplet. Traces show averages of 100 spectra each and are offset for clarity. (d) Intensity values for the microdroplet in (c), with no net change for nitrate and sulfate over time. WGM data are included to show WGM relative intensity.

Raman spectra of a glycine, nitrate, and sulfate microdroplet as a function of time are given in Fig. 4a. A pH change can be observed over the course of 82 minutes by monitoring the glycine peak intensities. The pH of the droplet is initially 1.8 and then reaches a pH of 2.4 during the course of the experiment. In addition, the nitrate peak decreases by 33% due to HNO_3_ partitioning out of the microdroplet and into the gas phase, while there is little change in the sulfate peak intensity (the intensities are plotted in Fig. 4b). To verify that this pH change is due to nitrate depletion and not occurring due to dilution of the droplet by the surrounding RH flow, we trapped a separate microdroplet composed only of sodium sulfate and sodium nitrate (pH unadjusted and estimated to be 5.4) for over 44 hours. During these 44 hours, there was no decrease in peak intensities as shown in Fig. 4c and d. Additionally, an expanded view of the first 82 minutes is shown in ESI Fig. S2.†

We also performed a nitrate depletion experiment with a microdroplet containing only sulfate and nitrate generated from a bulk solution at pH 1.7. This microdroplet exhibited nitrate depletion (see Fig. S3†). Therefore, we conclude that the total amount of acidity, rather than the presence of an organic acid (such as glycine) drives depletion. These results show that either inorganic or organic acids can serve as HA in eqn (1) and (2).

**Fig. 5:**
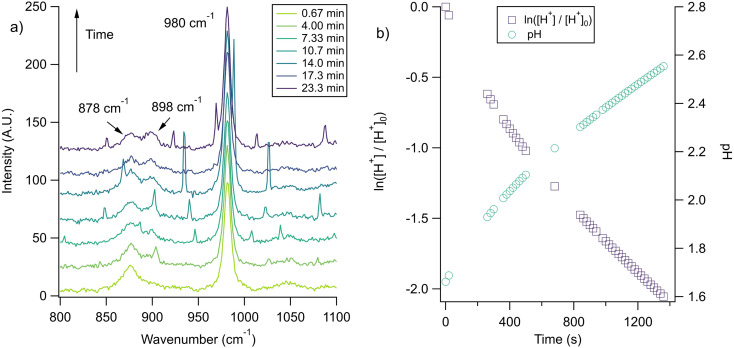
(a) A microdroplet was trapped from a solution of 2 m glycine and 1 m NaCl titrated to pH 1.7 using H_2_SO_4_. The ratio of the ratio of the intensities of glycine peaks, 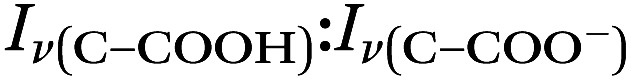, quickly changes, illustrating an increase in pH that corresponds to chloride depletion. The small, unmarked peaks are WGMs, characteristically identifiable using their changing locations in between spectra. The traces shown here are averages of 11 spectra each. (b) The change in pH over time for the same microdroplet (circles, right axis) as calculated using the intensity ratio of the glycine peaks and a first-order fit for the H^+^ concentration (squares, left axis).

The nitrate depletion results above demonstrate that either the Raman intensity of specific modes (in this case the symmetric stretch of the nitrate ion) can be directly probed for the species of interest or the pH of the microdroplet can be used to measure depletion. For chloride ion depletion, the latter is convenient because the chloride ion cannot be detected with Raman spectroscopy. Therefore, non-evaporating glycine and its use as a pH indicator can track chloride depletion in individual microdroplets. This is demonstrated in Fig. 5. As before, the initially acidic microdroplet increases in pH over time as indicated by the glycine Raman peak intensity ratio. The process is also faster compared to nitrate depletion, so the spectra were averaged over shorter time scales and fewer spectra making the WGMs more apparent.

**Fig. 6:**
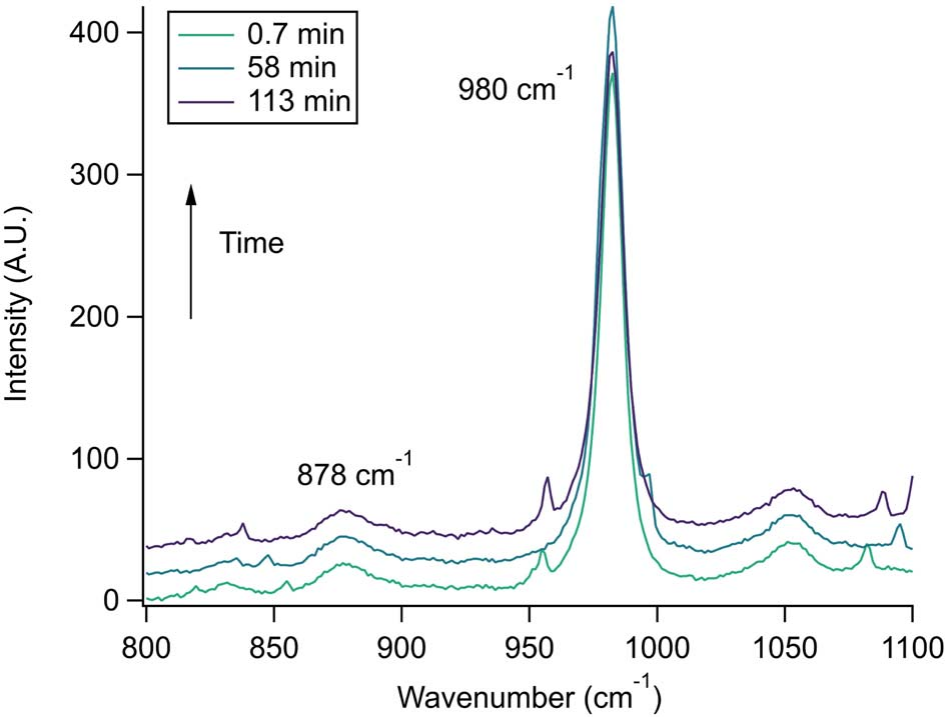
Control AOT experiment using a microdroplet created from a 1 m glycine 2 m Na_2_SO_4_ bulk solution acidified to pH 2 with sulfuric acid. No pH change was observed as evidenced by the constant intensity of the 878 cm^−1^ peak. Traces are offset for clarity and show averages of 120 spectra each, and small unmarked peaks correspond to WGMs.

Since we rely on the pH change shown by the glycine peaks to infer chloride depletion, it is important to verify that these changes do not occur due to other experimental factors. We tested this by trapping a microdroplet containing only glycine and sodium sulfate, with the Raman spectra shown in Fig. 6. No pH change is observed over *ca.* 2 hours, which is as expected since there is no chloride or nitrate available for depletion (initial pH was controlled with sulfuric acid). We note that no pH change is expected when RH is maintained constant as in the AOT; for substrate-deposited microdroplets in environments of changing RH, the glycine peak ratio may be impacted by the concentration or dilution even in the absence of depletion. To further demonstrate the utility of the glycine peaks in measuring chloride depletion, we performed an experiment with an initially neutral microdroplet, coalesced with an acidic HCl droplet at lower pH, and then observed a return to a higher pH as gas-phase HCl partitions from the microdroplet. These spectra are provided in ESI Fig. S4.†

These experiments were all carried out at 80% RH. Previous work on nitrate and chloride depletion has emphasized results for dehydrated droplets.^37^ Thermodynamically, depletion is driven by volatility and the partial pressure of HNO_3_ or HCl in the surrounding environment, so there is no strict theoretical requirement for dehydration.^38^ Even so, multiple factors promote more depletion when there is dehydration. These include acidification of the microdroplet, making the formation of HNO_3_ or HCl more likely, as well as concentration.^21^

A summary of AOT nitrate and chloride microdroplet depletion experiments is given in Table 1. Radii and refractive index *versus* time data for these experiments are given in Fig. S5.† After outliers corresponding to WGMs were removed (the impact of WGMs on intensity can be seen in Fig. 4d), peak intensities were monitored over time and fit to first-order kinetics in order to obtain rate constants, *k*, as previously described.^26^ The *k* values for chloride depletion are an order of magnitude larger than those for nitrate depletion. This is consistent with previous attributions of chloride depletion relative to nitrate.^39^ In addition, HCl is smaller than HNO_3_, so once transient HCl is formed, it may be able to more quickly diffuse to the interface and evaporate before dissociating again. Finally, there is evidence that chloride is enriched at the interface compared to the core, which would also make transient formation of HCl more likely.^40^ A larger absolute uncertainty in the *k* values for chloride depletion comes in part from the fact that the two glycine peaks, which overlap, must both be fitted to correctly track pH and from that a calculated rate, with each step adding to propagated error (see discussion in the ESI†).^41^

**Table 1:** Summary of single microdroplet depletion experiments and first-order rate constants *k*

Experiment type	Number of trials	*k* (s^−1^)	Microdroplet composition [initial pH]
NO_3_^−^ depletion (with glycine)	5	1.3 ± 0.4 × 10^−4^	NaNO_3_, Gly, Na_2_SO_4_ [1.3–1.7]
NO_3_^−^ depletion (no glycine)	1	1.9 ± 0.1 × 10^−4^	NaNO_3_, Na_2_SO_4_ [1.7^a^]
Cl^−^ depletion	3	1.0 ± 0.1 × 10^−3^	NaCl, Gly, Na_2_SO_4_ [1.4–1.7]
NO_3_^−^ control	1	N/A	NaNO_3_, Na_2_SO_4_ [5.4^a^]
Cl^−^ control	1	N/A	Gly, Na_2_SO_4_ [1.3]

a The pH of the bulk solution used since this microdroplet had no glycine for pH measurement.

### Comparison of the results of models and experiments

The kinetics of depletion have been theoretically derived by Chen *et al.*^38^ In that analysis, it was assumed that the depleting species (HNO_3_ or HCl) instantly formed at the air/water interface and then the rate of evaporation from a diffusion-limited mass-transfer model was calculated.^42^ The characteristic time, t*, and an associated constant, *k*, are given in eqn (7) and (8),7
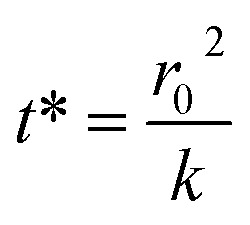
and8
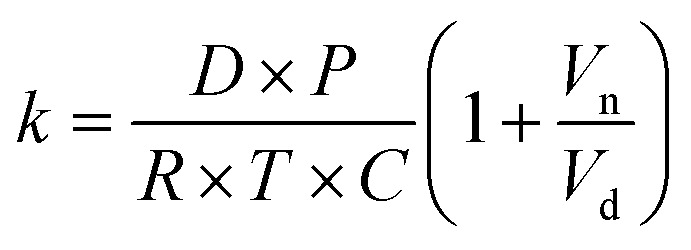
where *D* is the diffusion coefficient, *P* is the partial pressure at the microdroplet surface, *C* is the concentration of HCl or HNO_3_ in a droplet containing only the depleting solute, and *V*_n_ and *V*_d_ variables refer to the initial volume taken up by the nonvolatile and depleting species, respectively, while *R* is the gas constant, *T* is the temperature, and *r*_0_ is the initial microdroplet radius. A thermodynamic model such as the E-AIM is used to calculate *P*, *V*_n_ and *V*_d_ for a given system.

While this formulation yields useful insights on the upper limit rate at which these processes can occur, the rates of this process will likely proceed more slowly. For example, it has been previously shown that, while HNO_3_ is 20% more stable near the air–water interface compared to the bulk, its dissociation into ions occurs in approximately 0.3 nanoseconds.^23^ Therefore, rather than forming instantly in large quantities that diffuse to the surface for evaporation, it is likely that HNO_3_ or HCl occasionally forms transiently in the solution, and evaporation only occurs when formed at the air/water interface such that partitioning into the gas phase occurs before dissociation.

To put our results in context with this upper limit, we performed calculations with the model of Chen *et al.* and E-AIM using malonic acid in place of glycine (see Experimental methods).^38^ The purpose of the E-AIM calculation is to determine reasonable approximations for the partial pressure of the volatile species at the air–water interface and the volume occupied by the volatile and nonvolatile species in the microdroplet or aerosol. These parameters are used for the calculations below. Our E-AIM calculation used 2 moles malonic acid, 1 mol NO_3_^−^, 2.634 mol Na^+^, 1.235 mol SO_4_^2−^, and 0.836 mol H^+^. This yields a microdroplet with pH 2.1 at 80% RH and which has a species ratio similar to that in our experiments. The E-AIM results are otherwise not further analyzed due to the difficulties of implementing charge-balanced calculations with amino acids.

Using the aforementioned model, we calculated a characteristic depletion time of *ca.* 200 seconds for a 4 μm radius droplet. This is significantly faster than our measured rate constants, indicating that the kinetics we observed are not completely due to a diffusion limited process but instead also due to the probability of the depleting species associating near the interface. Our measured timescales also make more intuitive sense considering what has been observed previously for atmospheric aerosols. Aqueous atmospheric aerosols can remain airborne for a wide range of times, depending on size and wind among other factors, but often have lifetimes in the order of days.^43,44^ Given that total depletion is rarely observed for environmental aerosols, characteristic times of hours seem more reasonable than a few minutes. To place the results in terms of a concentration flux, we also performed a Maxwell droplet calculation for a 1 m HCl aerosol. Assuming an ambient concentration at an infinite distance from a droplet of 1 ppb, we obtain an HCl depletion flux of 43 nmol s^−1^.

More recently, Jing *et al.* showed an approach for tracking pH change and hence chloride depletion in mixed sodium chloride and oxalic acid droplets.^40^ Their data were reported as pH change over time, so in order to make a direct comparison, we analyzed their 80% RH data with first-order kinetics and obtained a chloride depletion rate constant of 7.0 ± 0.9 s^−1^ at 95% confidence for substrate-deposited aerosols with radii of *ca.* 9 μm. This rate is below what we found for both chloride and nitrate depletion, which we attribute to differences in the partial pressure of gases maintained above the droplets and the fact that their droplets were substrate-deposited, giving less surface area from which HCl could partition into the gas-phase. In addition, the droplets may be of different viscosities which will change partitioning rates as has been recently discussed.^28^ Furthermore, their findings are considerably slower than the theoretical rates discussed above, which they attribute to differences in partial pressures. Thus, this comparison highlights both the differences in depletion rates that can be obtained by different techniques and the importance of using experiments to demonstrate the extent to which theoretical predictions are representative of experimental measurements.

In other studies, rate measurements have also been recently reported for the depletion of ammonium.^45^ For this process, the pH of the solution decreases over time as NH_4_^+^ dissociates to release acidic protons and NH_3_, which can escape as a gas. In this case, the data were analyzed in terms of a characteristic lifetime *τ* using eqn (9).9
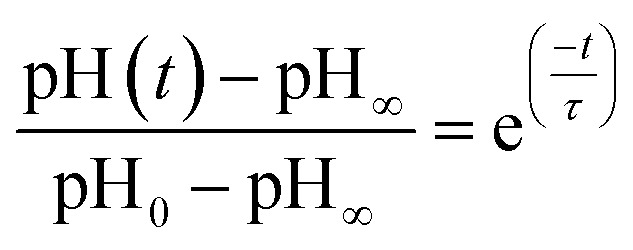
Here, pH(*t*) is the pH at a given time, pH_∞_ is the pH value the experiment approaches for long reaction times, pH_0_ is the initial pH, and *t* is time. At 80% RH, values of *τ* for malonic or succinic acid droplets ranged *ca.* 10 to 20 minutes for nanoparticle-containing droplets with average radii of 20 μm.^45^ Using a similar analysis on an experiment where we kept a microdroplet suspended for a protracted period of time, we calculated a *τ* of 8.6 minutes for chloride depletion. Once again, this comparison illustrates that the reactions proceed more slowly than the diffusion-limited rate predicted by Chen *et al.* thus showing the importance of experimental measurements in identifying differences and discrepancies between experiments and calculations and thus identifying the importance of different processes that lead to these differences.^38^

## Conclusions

Nitrate and chloride depletion rates from individual, levitated microdroplets have been measured using Raman spectral peak intensities and glycine as an *in situ* pH probe. First-order rate constants at 80% RH were found to be *ca.* 10^−3^ to 10^−4^ s^−1^. These rates indicate that appreciable depletion can be expected during the lifetime of an aqueous microdroplet or smaller aerosol. Even for rapidly evaporating secondary organic aerosols, which could lose half of their volume in less than 100 minutes, a loss of 60% of the initial nitrate could occur, leading to a net decrease in the concentration even with a decrease in volume.^46^ We emphasize that we have measured initial depletion rates here. As pH increases and nitrate or chloride concentrations decrease, the depletion rate would be even slower. Interestingly, if there are pH gradients with enhanced acidity at the air/water interface, the presence of H^+^ at the air–water interface could contribute to the formation and partitioning of HCl and HNO_3_ at the interface and then partitioning into the gas phase.^47^

We have also demonstrated that there are few requirements for depletion to take place and contest in some cases what has been suggested previously. We measured depletion at high relative humidity suggesting that dehydration is not a necessary requirement of the reaction and that both organic and inorganic acids can drive depletion. Therefore, we posit that total acidity is one of the key factors that drives depletion and not a particular acid (*e.g.* dicarboxylic acids). Although, because the volatility of dicarboxylic acids is low, similar to glycine, they will remain in the particle phase and provide acidic protons to contribute to nitrate or chloride for depletion.^48^ Acetic acid, on the other hand, is highly volatile and expected to be found predominantly in the gas phase.^34^ Therefore, acetic acid and similar low molecular weight monocarboxylic acids likely partition out of microdroplets and aerosols before appreciable depletion occurs. Our demonstration that glycine can cause depletion is useful because amino acids have been found to be greatly enriched in aerosols.^49,50^ It is also noteworthy because glycine is not highly surface active, demonstrating that the acid within the bulk, as described using eqn (1) and (2), does not have to reside at the surface.^4^ In addition, glycine offers the advantage of a relatively low viscosity (compared to oxalic acid, for example), allowing liquid-like microdroplets to be analyzed along with glassy and gel-like particles.^51–53^

Overall, our results show that pH changes in microdroplets can be monitored and the kinetics of these changes due to the depletion of acids from the microdroplet can be precisely determined. We project based on these studies that the limiting factor is formation of the depleting species, in this case a strong acid, in the vicinity of the air/water interface.

## Data availability

Data used for the creation of all figures in the manuscript are available in the ESI.†

## Author contributions

K. J. A. contributed to research design, experiments, analysis, and writing. V. H. G. contributed to research design, writing and editing.

## Conflicts of interest

The authors have no competing interests to declare.

## Supplementary Material

SC-014-D2SC06994F-s001
