# Supplementary material for: Direct quantification of changes in pH within single levitated microdroplets and the kinetics of nitrate and chloride depletion
Source: Chem Sci. 2023 Apr 11;14(23):6259–68. doi: 10.1039/d2sc06994f (PMC10266444; doi:10.1039/d2sc06994f)
Supplement: SC-014-D2SC06994F-s001 [file SC-014-D2SC06994F-s001.pdf]

Electronic Supplementary Material (ESI) for Chemical Science.
This journal is © The Royal Society of Chemistry 2023

Electronic Supplementary Information for:

Direct Quantification of Changes in pH within Single Levitated Microdroplets and the Kinetics of Nitrate and Chloride Depletion

Kyle J. Angle and Vicki H. Grassian*

Department of Chemistry and Biochemistry,

University of California San Diego, La Jolla, CA 92093

*Author to whom correspondence should be addressed (vhgrassian@ucsd.edu)

Microdroplet Composition Compared to the Composition of the Bulk Generating Solutions: Microdroplets that are trapped in the AOT can have different concentrations compared to the bulk solution that they are generated from. Most commonly is that the droplet concentrations can differ based on the amount of water loss between the nebulization and laser trapping process. For the glycine pH calibration, we determine the hydrogen ion concentration from the ratio of the two glycine peaks at 878 and 898 cm^{-1}, due to the cationic, fully protonated and zwitterionic forms, respectively, as discussed in detail in the main text. Thus both forms of glycine will be concentrated by the same amount since they are in the same microdroplet as neither form is volatile because they are ionic species and stay in the aqueous microdroplet. For nitrate or chloride depletion, we used first order kinetics to measure rates, which uses a plot with $[A]/[A_0]$, and is thus independent of initial concentration.

Supporting Figures

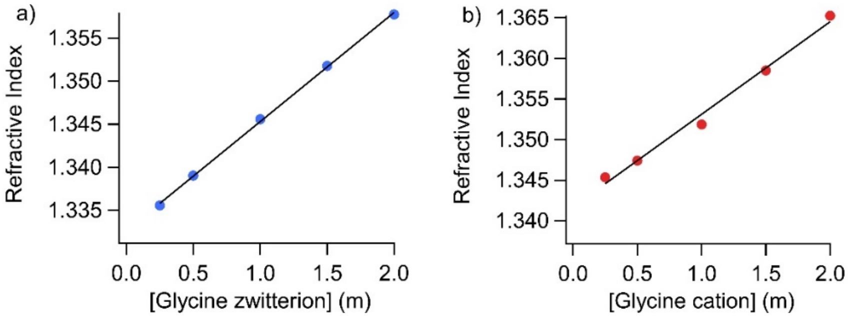

Figure S1. Refractive index data for glycine. (a) Refractive index for glycine solutions dissolved in pure water. (b) Refractive index for glycine solutions acidified with 6 N HCl such that > 99% of the glycine was cationic (ca. pH 0.6). Note that the intercept is higher than (a) due to the effect of the added acid.

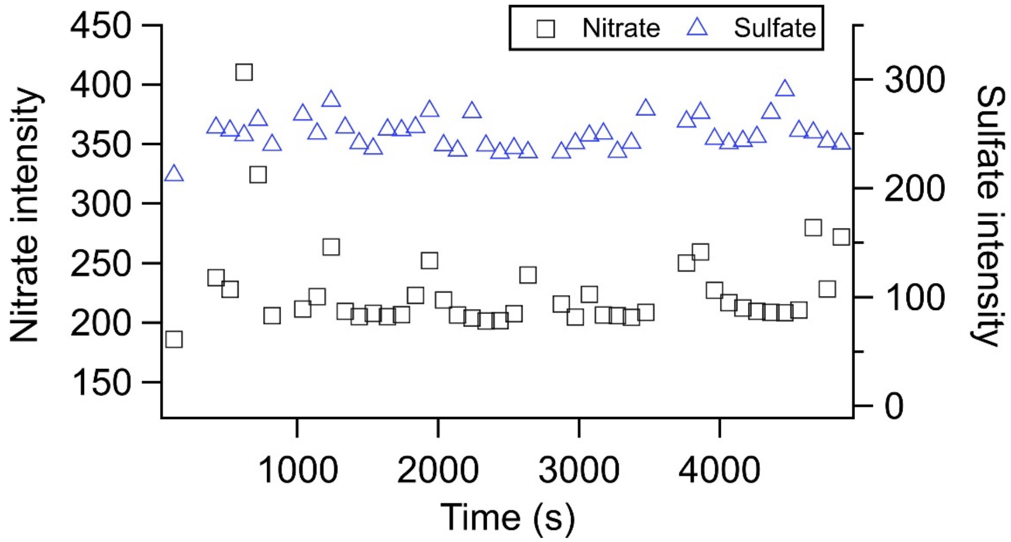

Figure S2. The first 80 minutes of the control experiment in Figure 4d is plotted. These data show that nitrate depletion doesn't appear happen on short or even on protracted timescales for non-acidic particles. Note the periodic bumps are from the WGMs.

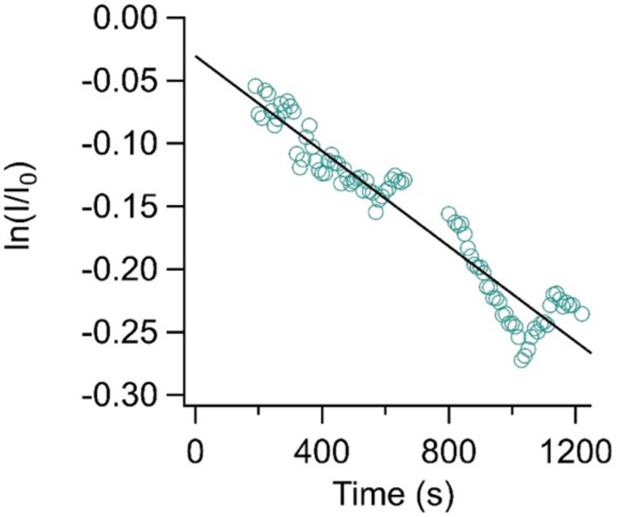

Figure S3: Nitrate depletion for a microdroplet without the presence of glycine or other organic compounds. A bulk solution of 1 m $NaNO_3$ and 1.2 m Na_2SO_4 adjusted to pH 1.7 with H_2SO_4 was prepared. The microdroplet was nebulized into a high RH environment (ca. 92 ± 8%) to reduce additional acidification from concentration. Spectra were collected for 152 minutes. During the first 21 minutes, nitrate depletion was observed via normalization of the nitrate peak to the sulfate peak. The first-order kinetics fit of this portion of the data is shown. The absolute value of the slope, which is *k*, is $1.9 \pm 0.1 * 10^{-4}$ s^{-1}, and R^2 is 0.92. While the pH of this microdroplet could not be directly measured due to the lack of glycine, the "pH probe" in this particular experiment. These data illustrate that depletion can occur in the absence of an organic compound such as glycine when there is sufficient acidity present.

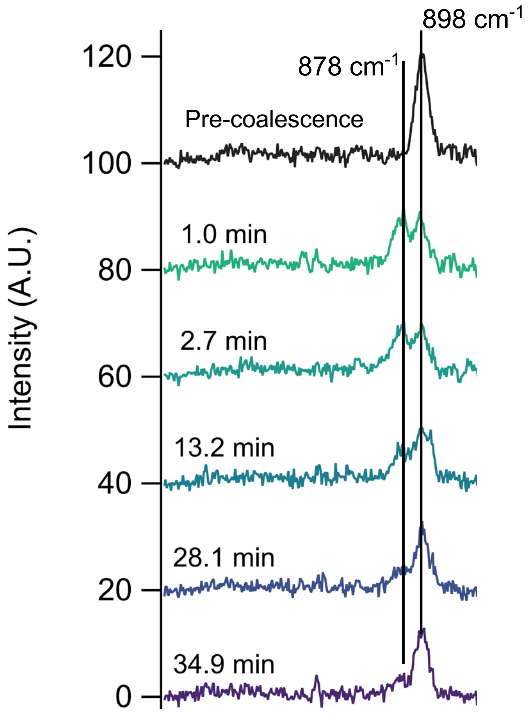

Figure S4: Changes in microdroplet acidity following a coalescence event. A microdroplet was trapped from a solution of 2 m glycine, 2 m NaCl and 0.1 m $NaNO_3$. The spectrum labeled "pre-coalescence" is recorded for the initially trapped droplet and is stabilized (ca. 8 minutes). The droplet is then titrated with a smaller 1 m HCl droplet through a droplet coalescence, as we have shown previously,[1] and thus acidified, at time = 0 minutes. The presence of the peak at 878 cm^{-1} due to ν(C-COOH) of the cationic, fully protonated form becomes apparent. Following this acidification of the microdroplet from the droplet coalescence event, gas-phase HCl partitions out of the microdroplet and de-acidifying the droplet in a multiphase buffering type of process which we have discussed recently.[2] Traces show averages of 10 spectra.

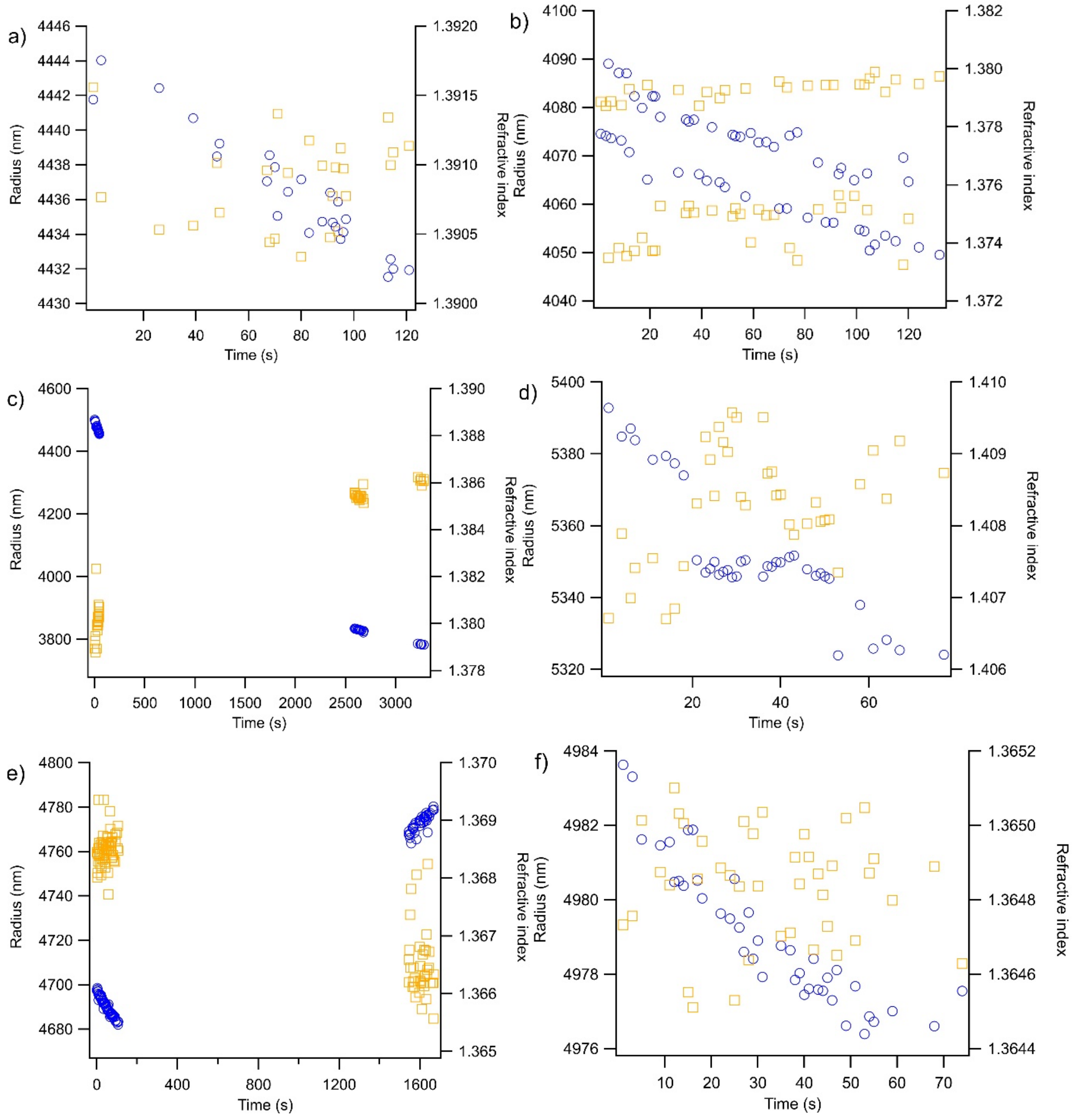
a)
Radius (nm)
Refractive index
Time (s)
b)
Radius (nm)
Refractive index
Time (s)
c)
Radius (nm)
Refractive index
Time (s)
d)
Radius (nm)
Refractive index
Time (s)
e)
Radius (nm)
Refractive index
Time (s)
f)
Radius (nm)
Refractive index
Time (s)

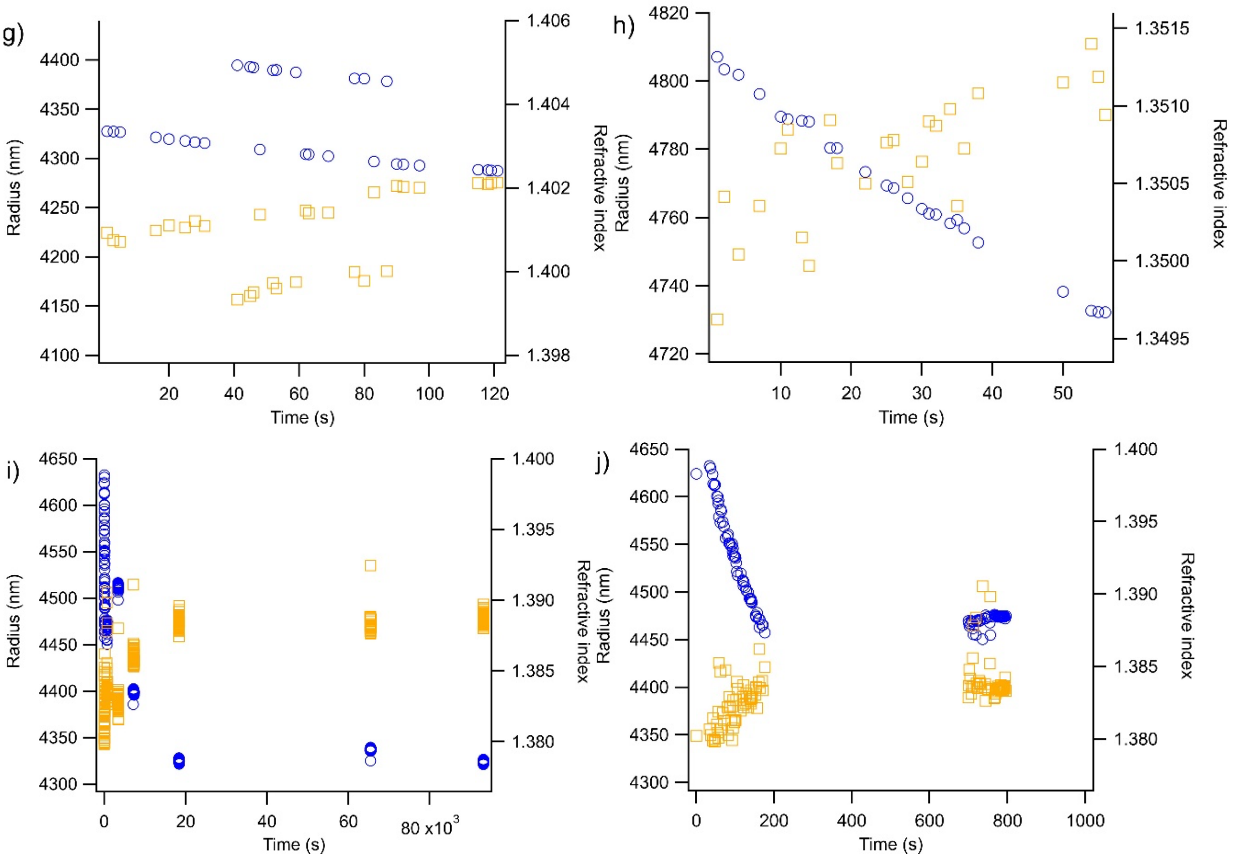

Figure S5: Additional radius and refractive index data. Plots a-d correspond to nitrate depletion experiments (row 1 of Table 1), e-g correspond to chloride depletion (row 3 of Table 1), and h-i correspond to nitrate depletion controls (no glycine) at low (h) and neutral (i&j) pH levels, (row 2 and 4 of Table 1, respectively; row 5 was not suitable for analysis due to the absence of NaCl and $NaNO_3$ which stabilize droplets within the laser trap). Note that the axes scales vary significantly between plots due to different experimental lengths and to more clearly show the spread of data. Generally, data can be unambiguously defined as data points where the WGMs are misidentified can be removed due to being physically unrealistic (for example, a refractive index less than that of water or a radius that suddenly jumps to 2000 nm in the middle of a 5000 nm trend). In plots b and g, two parallel traces were both physically realistic and thus both left in. Radii generally decrease with time, and the spread on the left hand side of plot i is shown in more detail in plot j, which presents the same data expanded. The exception is the right side of plot e, where an increase in radius may be due to an increase in chamber RH. Refractive index is consistently inversely correlated with radius (note the extreme magnification in plot f, the only plot where this is not visibly apparent) which is consistent with theoretical expectations.

Error Propagation

Here, we will analyze the error associated with the chloride depletion experiment with the most uncertainty to illustrate the scope of our method. At 95% confidence, the slope m of the calibration curve given in Figure 3 is 222 ± 6 m^{-1}, and the intercept b is 0.1 ± 0.1. For a particular chloride depletion microdroplet experiment, due to the overlap of the ν(C–COOH) and ν(C–COO$^-$) peaks, the average uncertainties in their peak intensities from Gaussian peak fitting are 10% and 19%, respectively. Terming the peak ratio y, based on Figure 3 we have

$$pH = -\log([H^+]) = -\log\left(\frac{y-b}{m}\right) \quad \text{(S1)}$$

Since y is determined from a ratio of peak intensities, we propagate its uncertainty via percentages. This uncertainty must then be converted back into an absolute uncertainty to propagate with b, which can be accomplished by using the average y value of 1.72 which we will term a_y, and then we once again convert to a percentage to propagate by division with m. Therefore, we get an error e according to the following:

$$e = \sqrt{\left[\frac{\sqrt{\left\{\sqrt{\left(\frac{s_{C-COOH}}{a_{C-COOH}}\right)^2 + \left(\frac{s_{C-COO-}}{a_{C-COO-}}\right)^2} * a_y\right\}^2 + s_b^2}}{a_y}\right]^2 + \left(\frac{s_m}{a_m}\right)^2} \quad \text{(S2)}$$

In this equation, s refers to the 95% confidence error of a given component, a refers to a given value (a slope, intercept, or average value from peak intensities) and different style brackets are used for visual clarity only (all work as parentheses). Above we reported 95% values as percentages for ease of reading, but taking s divided by a yields decimal answers, which are appropriate for error propagation.[3] For our high-error chloride depletion, e is 22%, which unsurprisingly is dominated by the uncertainty in the glycine peak intensities. As a point of comparison, the 95% confidence interval for the slope of the first-order kinetics fit for the same experiment is $1.05 \pm 0.06 * 10^{-3}$ s^{-1}, which carries an uncertainty of only 6%. In general, first-order fits were found appropriate for the initial depletion rates, with nitrate and chloride depletion having average R^2 values of 0.84 and 0.96, respectively. Finally, for the chloride depletion reported in Table 1, the standard deviation is 13%, which while within error propagation, may also be in part due to variability between trials from nebulization enrichment and hence different starting pH values. Overall, error propagation demonstrates the potential utility of future substrate-deposited experiments, where greater Raman signal will decrease the uncertainty in the overlapping glycine peaks, which currently dominate error for *individual* experiments. For uncertainty between different experiments, ideally enrichment would be better constrained, which could potentially be achieved with the development of new nebulization methods. As a final note, we used peak intensities instead of peak area in this study because we found both approaches gave similar average depletion k values, but using intensities led to significantly less uncertainty for chloride depletion. As long as the data points where a WGM directly overlaps with the peak of interest can be removed, using peak intensities gives a greater number of useful data points than peak areas, since in practice the travelling of WGM's across the small glycine C-COOH and C-COO$^-$ peaks led to substantial peak area uncertainty in our Igor fits. For chloride depletion, using peak intensities gave $k = 1.0 \pm 0.1 * 10^{-3}$ s^{-1}, while using peak areas gave $k = 1.4 \pm 0.8 * 10^{-3}$ s^{-1}. For nitrate depletion, the k values were $1.3 \pm 0.4 * 10^{-4}$ s^{-1} and $1.4 \pm 0.4 * 10^{-4}$ s^{-1}, respectively. Less error is found for nitrate depletion, likely because the Raman nitrate peak has a strong intensity and is well-defined for peak fitting.

Data Availability For All Figures in Main Text and ESI

Attached Excel file (DataPackage.xlsx).

References

(1) E. M. Coddens, K. J. Angle and V. H. Grassian, *J. Phys. Chem. Lett.*, 2019, **10**, 4476–4483.

(2) K. J. Angle, C. M. Nowak and V. H. Grassian, *Environ. Sci. Atmos.* 2023, **3**, 316–327.

(3) D. A. Skoog, S. R. Crouch and F. J. Holler, *Principles of Instrumental Analysis, 7th Edition*; 2016.
